# Recruiting and retaining medical patients in trials of complex interventions: practical experience of challenges and solutions in three multi-centre trials

**DOI:** 10.1186/1745-6215-12-S1-A127

**Published:** 2011-12-13

**Authors:** Jane Walker, Michael Sharpe

**Affiliations:** 1 Psychological Medicine Research, Department of Psychiatry, University of Edinburgh, Edinburgh, EH10 5HF, UK; 2 Psychological Medicine Research, Department of Psychiatry, University of Oxford, OX3 7JX, Oxford, UK

## Objectives

1. To consider the challenges of recruiting and retaining medical patients in trials of complex interventions, that include ‘psychological’ components. 

2. To discuss practical solutions to these challenges that ensure trials recruit to target and maximise outcome data collection. 

Examples are taken from three trials of complex interventions: SMaRT Oncology-2, SMaRT Oncology-3 and SMaRT Neurology-1.

## Methods

Challenges faced in the recruitment to three trials included: identifying eligible patients, achieving ‘buy-in’ from local clinicians, addressing the stigma associated with ‘psychological’ interventions and describing novel interventions to patients. Solutions included: identifying potential participants through systematic screening, minimising burden on clinicians, embedding the trial team in services and novel approaches to delivering trial information to patients. Challenges of retaining patients to ensure outcome data collection included: trial intervention withdrawals, patients’ physical deterioration and the collection of data at multiple time-points. Solutions included: ensuring a positive relationship between patients and the trial team, building relationships with local services and using financial incentives for patients.

Practical examples of each of these solutions are taken from the following trials: SMaRT Oncology-2 is a 500 patient cost-effectiveness trial comparing a complex intervention for depression in cancer patients to usual care, with the primary outcome at six months. SMaRT Oncology-3 is a 150 patient efficacy trial evaluating a similar intervention for depression in patients with lung cancer, who have limited life expectancy, with data collected over eight months to give an average depression score for the primary outcome. SMaRT Neurology-1 is a 130 patient efficacy trial of a guided self-help intervention for patients with medically unexplained neurological symptoms, with a primary outcome at 3 months.

## Results

All three trials recruited successfully and have retained sufficient participants to ensure high quality outcome data are recorded.

## Conclusion

Recruiting and retaining medical patients in trials of complex ‘psychological’ interventions poses special challenges. Innovative practical solutions can ensure recruitment to target and collection of adequate outcome data.

